# Assessing the impact of mRNA vaccination in chronic inflammatory murine model

**DOI:** 10.1038/s41541-024-00825-z

**Published:** 2024-02-15

**Authors:** Seonghyun Lee, Jisun Lee, Sun-Hee Cho, Gahyun Roh, Hyo-Jung Park, You-Jeung Lee, Ha-Eun Jeon, Yu-Sun Lee, Seo-Hyeon Bae, Sue Bean Youn, Youngran Cho, Ayoung Oh, Dahyeon Ha, Soo-Yeon Lee, Eun-Jin Choi, Seongje Cho, Sowon Lee, Do-Hyung Kim, Min-Ho Kang, Mee-Sup Yoon, Byung-Kwan Lim, Jae-Hwan Nam

**Affiliations:** 1 Department of Medical and Biological Sciences, The Catholic University of Korea, Gyeonggi-do, Bucheon, Republic of Korea; 2 BK21 four Department of Biotechnology, The Catholic University of Korea, Gyeonggi-do, Bucheon, Republic of Korea; 3 Department of Health Sciences and Technology, GAIHST, Gachon University, Incheon, 21999 Republic of Korea; 4 Department of Biomedical Science, Jungwon University, Goesan-gun, Chungbuk, 28024 Republic of Korea; 5 SML Biopharm, Gwangmyeong, 14353 Republic of Korea; 6 Department of Biomedical-Chemical Engineering, The Catholic University of Korea, 43 Jibong-ro, Bucheon-si, Gyeonggi-do 14662 Republic of Korea; 7 Department of Molecular Medicine, College of Medicine, Gachon University, Incheon, 21999 Republic of Korea; 8 Lee Gil Ya Cancer and Diabetes Institute, Incheon, 21999 Republic of Korea

**Keywords:** RNA vaccines, Chronic inflammation

## Abstract

The implications of administration of mRNA vaccines to individuals with chronic inflammatory diseases, including myocarditis, rheumatoid arthritis (RA), and inflammatory bowel disease (IBD), are unclear. We investigated mRNA vaccine effects in a chronic inflammation mouse model implanted with an LPS pump, focusing on toxicity and immunogenicity. Under chronic inflammation, mRNA vaccines exacerbated cardiac damage and myocarditis, inducing mild heart inflammation with heightened pro-inflammatory cytokine production and inflammatory cell infiltration in the heart. Concurrently, significant muscle damage occurred, with disturbances in mitochondrial fusion and fission factors signaling impaired muscle repair. However, chronic inflammation did not adversely affect muscles at the vaccination site or humoral immune responses; nevertheless, it partially reduced the cell-mediated immune response, particularly T-cell activation. These findings underscore the importance of addressing mRNA vaccine toxicity and immunogenicity in the context of chronic inflammation, ensuring their safe and effective utilization, particularly among vulnerable populations with immune-mediated inflammatory diseases.

## Introduction

mRNA vaccines have induced a paradigm shift in immunization, effectively preventing severe illnesses and reducing mortality rates during the coronavirus disease 2019 (COVID-19) pandemic^1,2^. However, the safety profile of mRNA vaccines in individuals with chronic inflammatory diseases must be considered. Understanding the potential risks and evaluating the safety of mRNA vaccines in the presence of chronic inflammation is paramount for ensuring the safe administration of these vaccines to vulnerable populations^3,4^. Specifically, an elevated risk of myocarditis associated with mRNA vaccines was observed in individuals aged under 40, emphasizing the importance of age-related considerations in vaccine safety assessments^5^. Chronic inflammatory diseases, such as obesity, rheumatoid arthritis (RA), systemic lupus erythematosus, and inflammatory bowel disease (IBD), are characterized by dysregulated immune responses and persistent inflammation^6–11^. Although the implications of the administration of mRNA vaccines to individuals with chronic inflammatory diseases are unclear, recent studies reported intrathecal inflammation in multiple sclerosis^12^ and acceleration of RA disease activity in chronic eosinophilic pneumonia after COVID-19 mRNA vaccination^13^. Generally, individuals with chronic inflammatory diseases receive immune-modulatory agents. However, recent reports showed that individual immune-modulatory treatments are not associated with the decreased immune response of SARS-CoV-2 vaccines^14^. These conditions are associated with theoretical risks of immune dysregulation and disease activity exacerbation following mRNA vaccination. In particular, the effectiveness of mRNA vaccines is reportedly reduced in obese individuals. Although obesity represents low-grade chronic inflammation, examining the effects of vaccines in these chronic inflammation models is necessary to rule out the impact of various factors involved in obesity. In addition to comprehensively assessing the safety of mRNA vaccines against chronic inflammation, investigating their potential effects on specific organs and tissues is important.

Acute myocarditis is an inflammatory disease of the heart muscle, wherein patients experience viral prodromes of fever, rash, myalgia, arthralgia, fatigue, and respiratory or gastrointestinal symptoms^15^; these nonspecific complaints typically precede cardiovascular symptoms by a few days to weeks^16^. Its clinical presentation is variable, and symptoms range from mild dyspnea or chest pain that resolves spontaneously to arrhythmias and cardiogenic shock^17^. In one case series, myocarditis was identified as the cause of unexplained dilated cardiomyopathy in 9.6% of the cases^18^. Several epidemiological studies that enrolled hospitalized patients with myocarditis before the COVID-19 pandemic have shown that the global incidence of this disease is ~1–10 cases per 100,000 people annually^19^, with the highest incidence in adolescents and young adults^20^. However, after the COVID-19 pandemic in 2020, the incidence of myocarditis significantly increased worldwide^21^. The risk of myocarditis in the general population has increased after the administration of the COVID-19 vaccine, but is still lower than that caused by SARS-CoV-2 infection^22^. Thus, a more comprehensive understanding of the potential risks and underlying mechanisms is needed to provide evidence-based information for facilitating informed decision-making regarding mRNA vaccine administration to this susceptible population.

In this study, we aimed to evaluate the safety of mRNA vaccines, with a specific focus on cardiac muscular toxicities, including myocarditis and pericarditis and muscle-related side effects at the vaccine injection site, using an in vivo model of chronic inflammation induced by an implanted pump system releasing bacterial lipopolysaccharide (LPS).

## Results

### Physicochemical characterization of mRNA vaccine

**Fig. 1:**
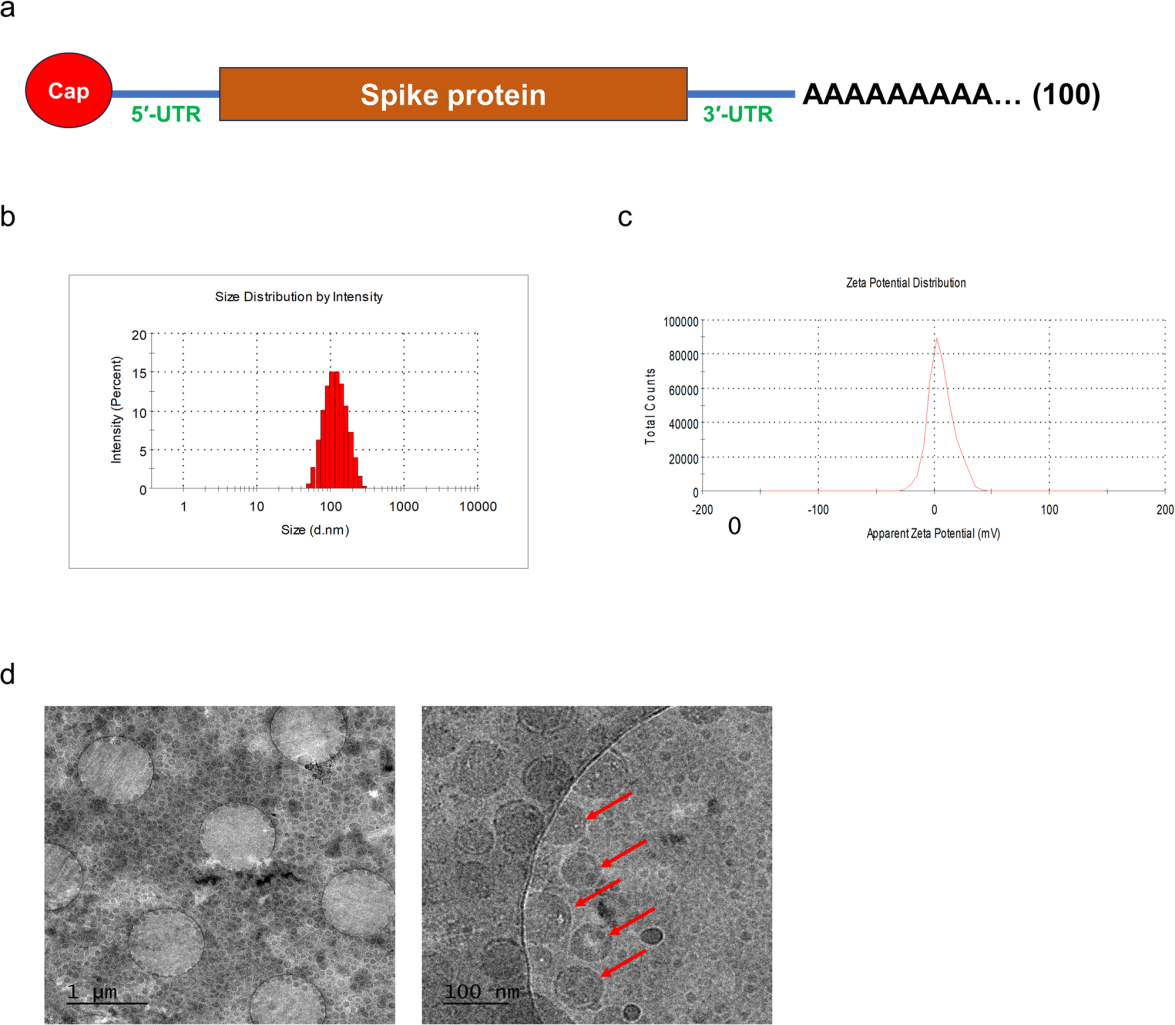
The physicochemical properties. **a** The design of the mRNA vaccine used in this experiment. **b**, **c** The size and zeta potential of the lipid nanoparticle comprising the mRNA vaccine. **d** Images of the lipid nanoparticle taken by Cryo-TEM. Each scale bars indicate 1 mm, 100 μm (left, right).

The mRNA vaccine utilized for immunization comprised the region located between the 5′ and 3′-UTR of the mRNA encoding the spike protein of the Omicron variant. It was formulated with lipid nanoparticles (LNPs) for mRNA stability and delivery (Fig. 1a). The Z-average size of the LNP measured 109.9 nm, with a zeta potential of 6.2 mV (Fig. 1b, c). Cryo-TEM images illustrated the stable formulation of mRNA with LNP, exhibiting uniform features (Fig. 1d). Consequently, the mRNA vaccine employed in this experiment was uniformly formulated with LNPs and maintained a stable physicochemical structure (Fig. 1).

### Development of a mouse model of LPS-induced chronic inflammation

**Fig. 2:**
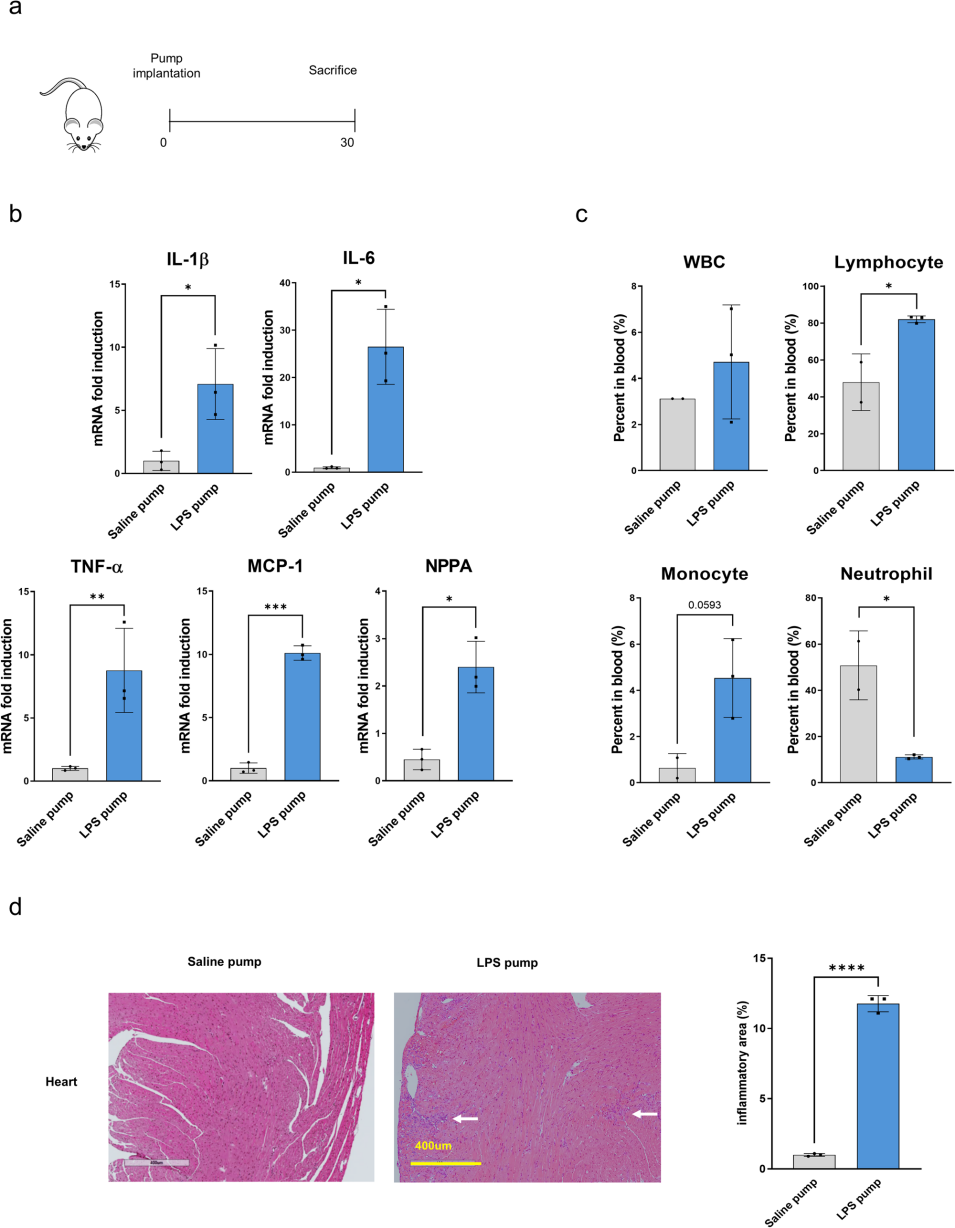
Generation of a chronic inflammation mouse model. **a** Schematic diagram of the animal experiment. A lipopolysaccharide (LPS) pump was implanted for 30 days, and the mice were subsequently sacrificed to collect tissue samples for analysis. **b** RNA was extracted from the heart of groups G1 and G2, followed by quantitative reverse transcription-PCR for interleukin-1 beta, interleukin-6, tumor necrosis factor alpha, monocyte chemoattractant protein-1, and natriuretic peptide A (*n* = 3 each group). **c** Peripheral blood was subjected to hematological analysis. White blood cell, lymphocyte, monocyte, and neutrophil numbers were counted and presented as blood percentages. **d** Histologic findings showed focal inflammatory cell infiltration in the hearts of LPS pump-implanted mice (white arrow). Scale bars indicate 400 μm. The percent area of inflammation was quantified from three or four fields of each heart. All data were presented as the mean ± standard deviation of values from independent experiments. NS >0.05, *P < 0.05, **P < 0.01, and ***P < 0.001 by a two-tailed Student’s *t*-test. IL-1β, interleukin-1 beta, IL-6, in*t*erleukin-6, LPS, lipopolysaccharide, WBC, white blood cell, TNF-α, tumor necrosis factor alpha, MCP-1, monocyte chemoattractant protein-1, NPPA, natriuretic peptide A.

To induce chronic inflammation in mice, LPS was delivered at a constant rate (0.11 μg/h) through a subcutaneously embedded LPS pump over 4 weeks. A pump infused with saline served as the control. Thirty days after pump implantation, the heart, liver, and pancreas were collected to assess inflammation and histological changes (Fig. 2a). The LPS pump induced mild and persistent inflammation in the heart. The mRNA levels of inflammatory cytokines, such as interleukin-1 beta, interleukin-6, interleukin-18, and TNF-α, were significantly higher in the LPS pump group than in the saline pump group. Additionally, the levels of monocyte chemoattractant protein-1 (a macrophage infiltration marker) and natriuretic peptide A (a cardiovascular damage marker) were significantly increased in the LPS pump group (Fig. 2b). Hematological analysis of whole blood revealed increased white blood cells, lymphocytes, and monocytes in the LPS pump group. However, chronic inflammation led to a reduction in the number of neutrophils (Fig. 2c). Furthermore, significant inflammatory cell infiltration was observed after hematoxylin and eosin staining of heart cross-sections (Fig. 2d), whereas no histological differences were observed in the liver and pancreas (Supplementary Fig 1a). Overall, a model of chronic inflammation was successfully established by subcutaneously implanting an LPS pump, and the inflammatory response induced by this model was primarily observed in the heart.

### mRNA vaccine administration exacerbates cardiac damage in chronic inflammation

**Fig. 3:**
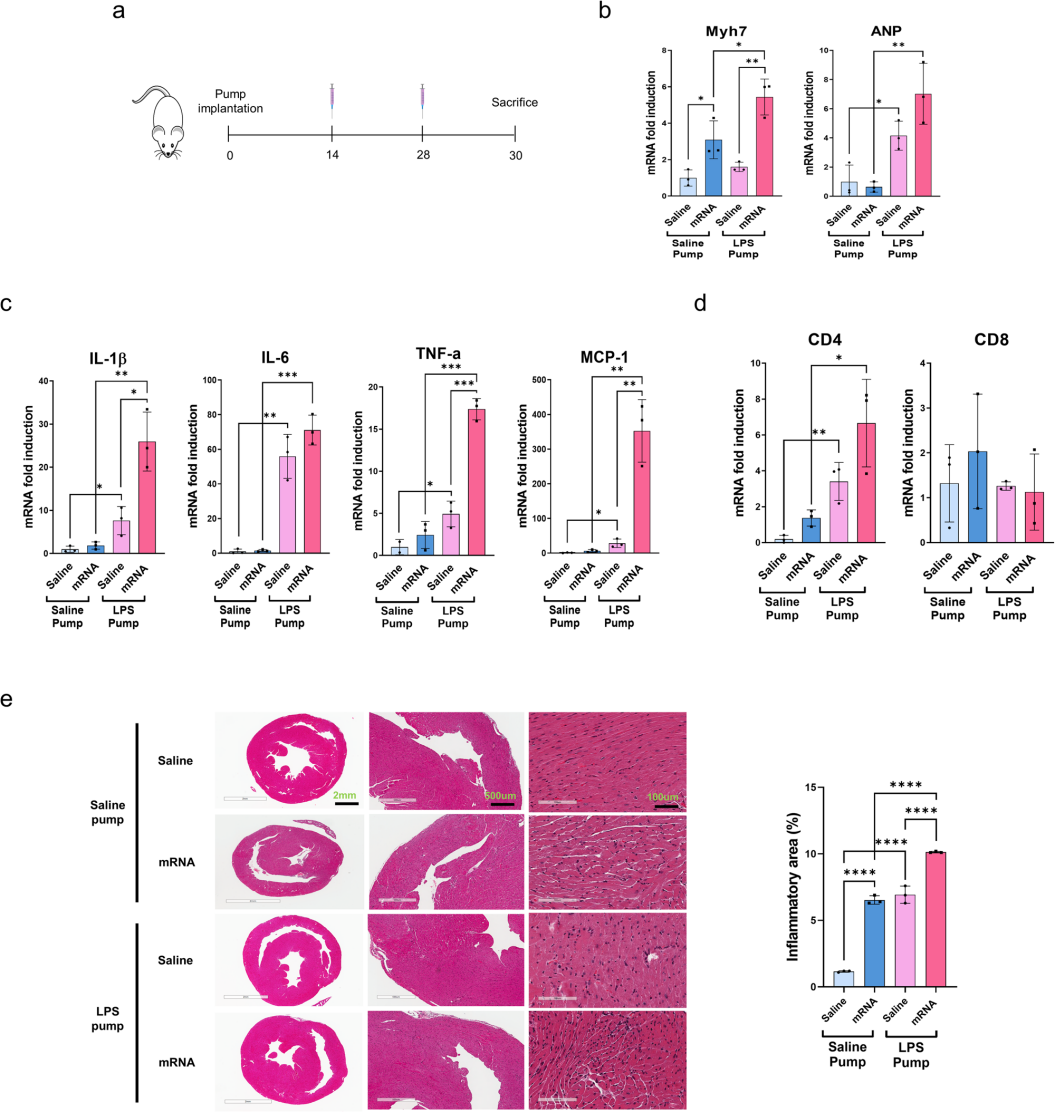
Administration of the mRNA vaccine in a mouse model of chronic inflammation. **a** Schematic diagram of the animal experiment. **b**–**d** RNA was extracted from the hearts of the mice in each group (*n* = 3 per group). The mRNA levels of heart damage markers (myosin heavy chain-7 and atrial natriuretic peptide), inflammation markers (interleukin-1 beta, interleukin-6, tumor necrosis factor alpha, and monocyte chemoattractant protein-1), and T-cell markers (CD4 and CD8) were confirmed using quantitative reverse transcription-PCR. **e** The heart inflammation level was observed using hematoxylin and eosin staining. Each scale bars indicate 2 mm, 500 μm, 100 μm (left, mid, right). The inflammatory percent area was quantified from three or four fields of each heart using NIH-ImageJ software. All data were presented as the mean ± SD of values from independent experiments. NS >0.05, *P < 0.05, **P < 0.01, and ***P < 0.001 by a two-tailed Student’s *t*-test. Myh7, myosin heavy chain-7, ANP, atrial natriuretic peptide, IL-1β, interleukin-1 beta, IL-6, interleukin-6, LPS, lipopolysaccharide, TNF-α, tumor necrosis factor alpha, MCP-1, monocyte chemoattractant protein-1.

To investigate the effect of the mRNA vaccine in the presence of chronic inflammation, the mRNA vaccine was intramuscularly administered on days 14 and 28 after LPS pump implantation (Fig. 3a). Quantitative reverse transcription-PCR analysis showed that the mRNA expression of myosin heavy chain-7, a cardiac hypertrophy marker, significantly increased in the heart tissue of the mRNA-administered groups with or without the LPS pump. Furthermore, atrial natriuretic peptide mRNA levels, which indicate increased plasma volume under high blood pressure and heart failure^23^, were significantly higher in the LPS pump-implanted groups than in the saline pump group. In particular, the mRNA vaccine amplified myosin heavy chain-7 and atrial natriuretic peptide levels much higher than those in the LPS pump group (Fig. 3b). Furthermore, the transcription of inflammatory cytokines and macrophage activity genes (interleukin-1 beta, interleukin-6, TNF-α, and monocyte chemoattractant protein-1) was significantly increased in the LPS pump with mRNA vaccine group compared to that in the saline-administered control group (Fig. 3c). Moreover, administration of the mRNA vaccine slightly enhanced the expression of CD4 and CD8, which are cellular immune response markers (Fig. 3d). Histopathological analyses of the heart 2 days after the second vaccine dose showed that the percentage of the inflammatory area was significantly increased by mRNA vaccine immunization in the LPS pump-implanted group. Notably, myocarditis was only induced by mRNA vaccine immunization (Fig. 3e). The liver, pancreas, and spleen showed no histopathological differences between the mRNA-vaccinated and unvaccinated LPS pump-implanted groups (Supplementary Figure 2a). These results demonstrate that mRNA vaccine immunization induces cardiac damage and myocarditis under chronic inflammatory conditions.

### Administration of mRNA vaccines does not induce significant adverse effects in muscles regardless of chronic inflammation

**Fig. 4:**
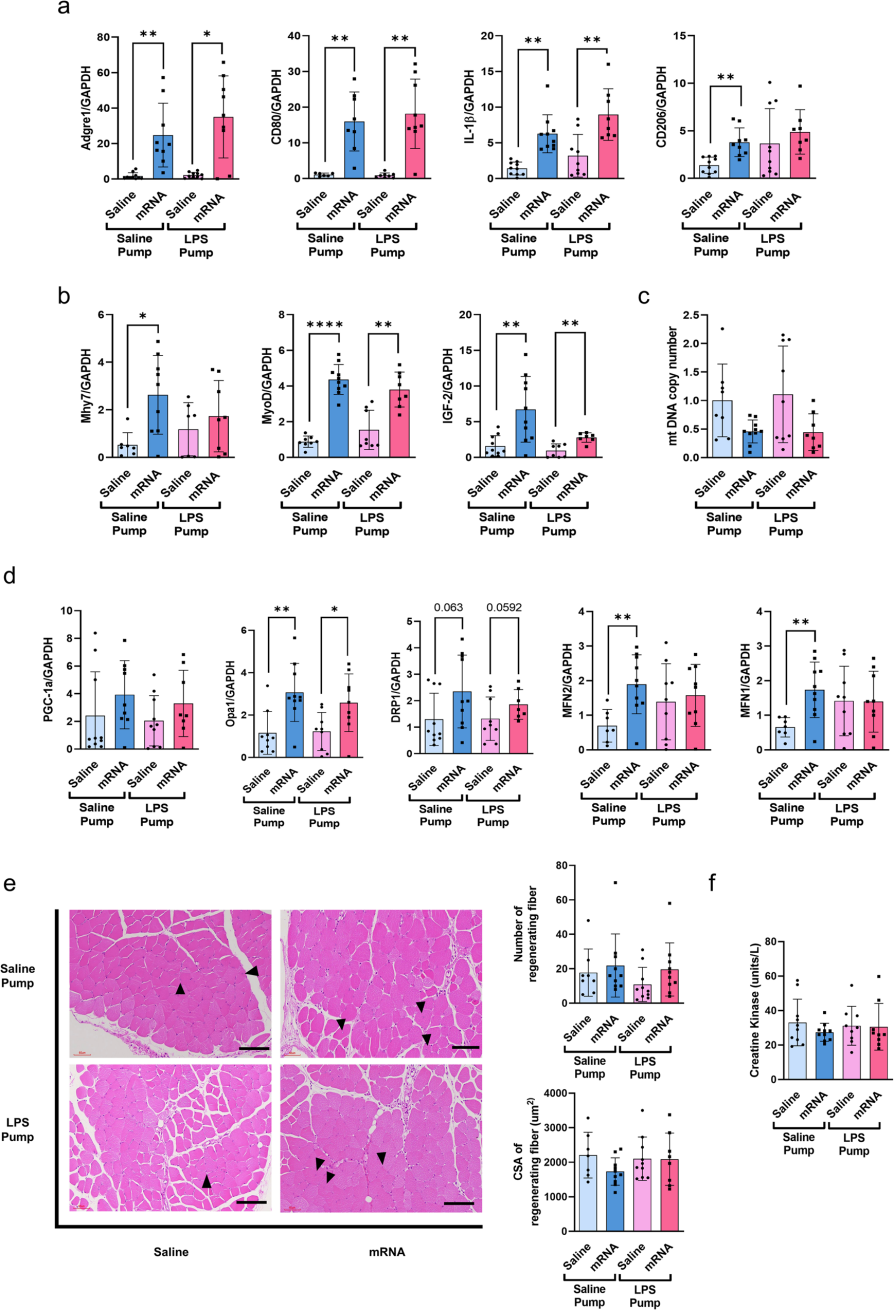
Non-impact mRNA vaccines on muscle tissue irrespective of chronic inflammation. **a**, **b**, **d** Quadricep muscles were collected two days after boosting immunization and analyzed using quantitative reverse transcription-PCR (*n* = 7–10). Mouse *GAPDH* was employed as the reference gene to standardize the expression levels of target genes. **c** The ratio between the copy numbers of mitochondrial DNA and nuclear DNA was determined by comparing the expression of NADH dehydrogenase subunit 1 with that of hexokinase 2. **e** Representative hematoxylin and eosin images of muscles from each group are shown. The scale bar represents 60 µm. The numbers and cross-sectional area of all centrally nucleated regenerating myofibers in the quadricep muscles were determined. **f** Serum creatine kinase activity was measured following the manufacturer’s protocol. *P < 0.05 **P < 0.01 by a two-tailed Student’s t-test. vs. the saline pump group or mRNA vaccination group. All data were presented as mean ± standard deviation. IL-1β, interleukin-1 beta, Myh7, myosin heavy chain-7, PGC-1α, peroxisome proliferator-activated receptor-gamma coactivator 1 alpha, Opa1, optic atrophy 1, Drp1, dynamic-related protein 1, MFN2, mitofusin 2, MFN1, mitofusin 1, LPS, lipopolysaccharide, CSA, cross-sectional area.

Administration of the mRNA vaccine increased the mRNA levels of Adgre1, indicating increased macrophage infiltration into muscle tissues (Fig. 4a). Similarly, mRNA vaccination resulted in elevated expression of both M1 markers, CD80 and interleukin-1 beta, and the M2 marker CD206 (Fig. 4a). However, no significant alteration was observed in the LPS pump-implanted group, wherein LPS increased CD206 expression in response to mRNA vaccine immunization. Next, we assessed the expression of factors associated with muscle satellite cell differentiation. Satellite cells, a group of skeletal muscle stem cells, are activated in response to injury, inducing their migration to the damaged site and subsequent proliferation, differentiation, and eventual fusion with injured muscle fibers^24^. Regardless of LPS pump implantation, the immunized group exhibited increased expression of the differentiation markers myosin heavy chain-7, MyoD, and IGF-2 (Fig. 4b). However, the increase in the expression of these factors was less pronounced in the LPS pump-implanted group than in the non-implanted group. Furthermore, no significant changes were observed in the ratios of muscle types, as evidenced by the unchanged ratios of TNNC1/TNNC2 and TNNI1/TNNI2 (Supplementary Figure 3a). Previous studies have demonstrated the detrimental effects of impaired mitochondrial dynamics on muscle mass and function^24,25^. The mitochondrial DNA content decreased after mRNA vaccine immunization, regardless of LPS pump implantation (Fig. 4c). However, the expression of peroxisome proliferator-activated receptor-gamma coactivator 1-alpha, a crucial regulator of mitochondrial biogenesis, showed a slight but insignificant enhancement in the mRNA-vaccinated groups compared to that in the unimmunized groups (Fig. 4d). Mitochondrial fission and fusion are controlled by specific proteins involved in mitochondrial dynamics^16^. Notably, the expression levels of fusion regulators, including optic atrophy 1, mitofusin 1 and 2, and fission regulator dynamic-related protein 1, were significantly higher in the mRNA-vaccine-immunized group than in the non-implanted group. These differences were not observed in the LPS pump-implanted group (Fig. 4d). Hematoxylin and eosin staining of quadricep muscle cross-sections revealed a slight increase in regenerating myofiber cells, characterized by a centrally localized nucleus, in the mRNA vaccine-immunized groups (Fig. 4e). However, no significant changes were observed in the quantity and cross-sectional area of the regenerated fibers or the number and size of blood vessels (Fig. 4e and Supplementary Figure 3b). Consistent with these results, creatine kinase activity, an indicator of muscle damage or injury, remained unchanged following LPS pump implantation and mRNA vaccination (Fig. 4f).

### Administration of mRNA vaccines reduced T-cell activation in a chronic inflammatory mouse model

**Fig. 5:**
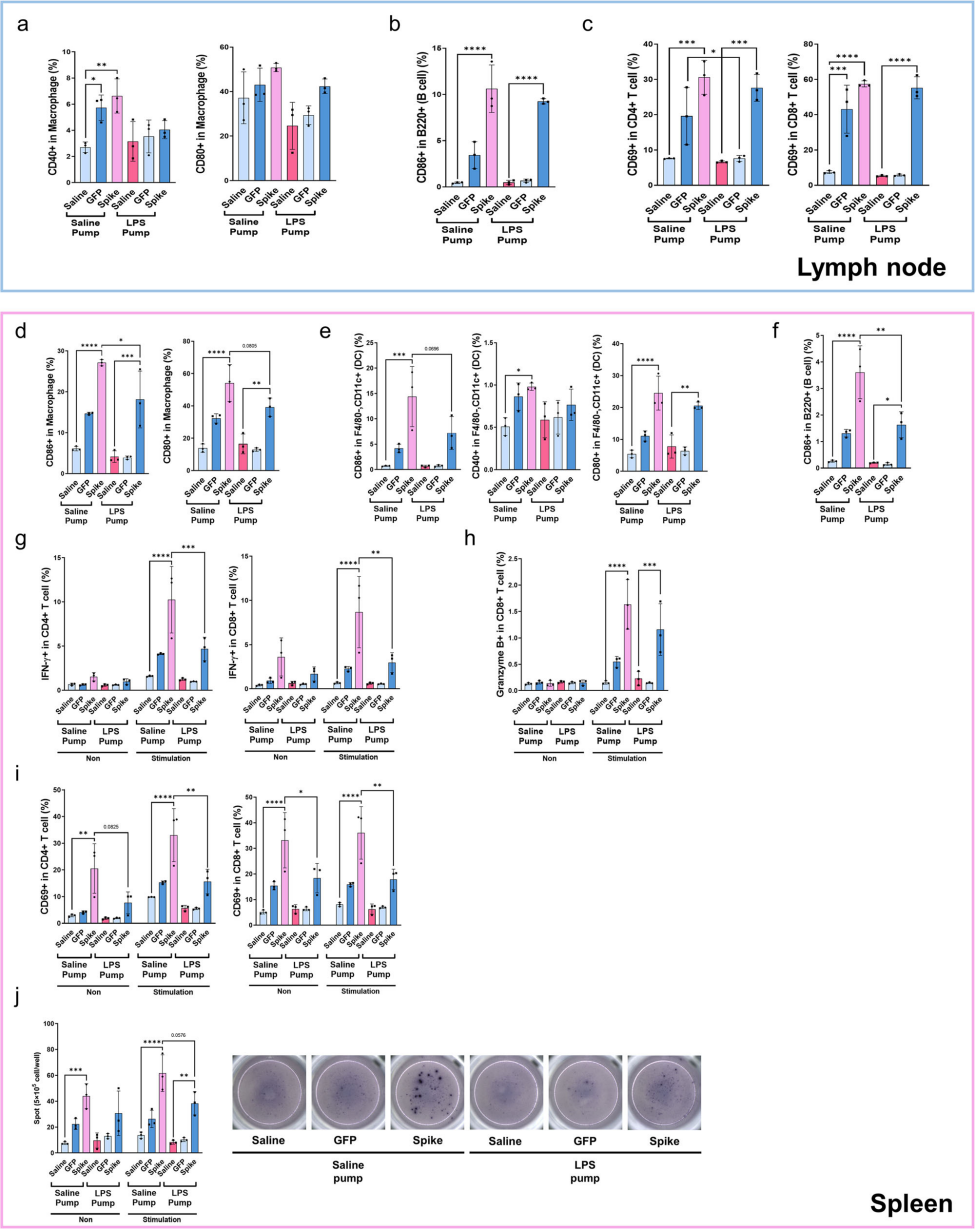
Effects of chronic inflammation on the immunogenicity of mRNA vaccines. C57BL/6 mice (*n* = 3) implanted individually with saline and LPS pump were intramuscularly primed and boosted with saline, lipid nanoparticle-formulated mRNA-Green fluorescent protein (GFP), and mRNA-Omicron (10 μg) at 2-week intervals and were subsequently sacrificed 2 days after boosting. Lymph nodes and splenocytes were isolated and analyzed with ELISpot assay and flow cytometry. **a**, **b** The activation levels in macrophages and B cells in lymph nodes were analyzed with flow cytometry. **c** The activation levels in CD4 and 8 T cells in lymph nodes were analyzed with flow cytometry. **d**–**f** The activation levels in macrophages, dendritic cells, and B cell in splenocytes were analyzed with flow cytometry. **g**, **h** Interferon-gamma (IFN-γ) positive CD4, CD8 T cells in splenocytes were stimulated with with/without Omicron-specific T-cell peptide (5 μg/ml) for 12 h at 37 °C and analyzed with flow cytometry. **i** The activation levels in CD4, 8 T cells in splenocytes were stimulated with/without Omicron-specific T-cell peptide (5 μg/ml) for 12 h at 37 °C and analyzed with flow cytometry. **j** Enzyme-linked immunospot (ELISpot) assay for interferon-gamma (IFN-γ) produces splenocyte activity. Splenocytes were stimulated for 2 days with/without Omicron-specific T-cell peptide (5 μg/ml). The IFN-γ secreting cell numbers were determined using ELISpot. *P < 0.05, **P < 0.01, ***P < 0.005 by a two-tailed Student’s t-test (**a**– **f**) or two-way ANOVA (**g**–**j**). vs. the saline pump or mRNA vaccination group. All data were presented as mean ± standard deviation. IFN-γ, interferon-gamma, LPS, lipopolysaccharide.

We isolated and analyzed lymph nodes and splenocytes using ELISpot and flow cytometry to evaluate the overall immunogenicity of the mRNA vaccine in a chronic inflammatory mouse model. In mice immunized with the GFP or Spike encoding mRNA vaccine (Fig. 5), we observed significant increases in the levels of CD40, which are markers of macrophages among individuals implanted with the saline pump compared to those implanted with the LPS pump. Similarly, on comparing groups receiving the same protein-encoding mRNA, with either saline or LPS pumps, we noted a slight elevation in CD80 marker expression in saline pump-bearing mice compared to that in LPS pump-bearing mice. However, these differences were not significant (Fig. 5a). B cells in the saline pump group with the GFP encoding mRNA vaccine displayed higher CD86 (marker) expression than those in the LPS pump. However, no significant difference was observed between Spike mRNA groups, regardless of pump type (Fig. 5b). CD4 and CD8 T-cell activation patterns closely resembled those in Fig. 5b (Fig. 5c). Specifically, the saline pump group with the GFP encoding mRNA vaccine exhibited increased CD4 and CD8 activation compared to the LPS pump group, while no significant difference was observed between Spike mRNA groups, irrespective of the pump used (Fig. 5c). We found no differences in dendritic cell (DC) activation and IFN-γ secretion levels between Saline and LPS pump groups (Figure S4a, b). In the spleen, a significant decrease in the activation of antigen-presenting cells (APCs), including macrophages, DCs, and B cells, was observed in mice implanted with the LPS pump and administered the Spike-encoding mRNA vaccine compared to those implanted with the Saline pump (Fig. 5d–f). Further, when splenocytes were stimulated with spike protein peptides, the number of IFN-γ-positive CD4 and CD8 T cells significantly decreased in the Spike mRNA vaccine with the LPS pump group (Fig. 5g). Additionally, while granzyme B levels, reflecting CD8 T-cell cytotoxic activity, exhibited a slight decrease in the Spike mRNA vaccine with the LPS pump group, this was not significant (Fig. 5h). Moreover, LPS pump-bearing mice immunized with the Spike encoding mRNA vaccine showed significantly lower CD4 and CD8 T-cell activation than those with the saline pump (Fig. 5i). Furthermore, IFN-γ secretion from splenocytes stimulated with spike protein peptides was significantly reduced in LPS pump-bearing mice immunized with the Spike encoding mRNA vaccine compared to Saline pump-bearing mice (Fig. 5j). Subsequent experiments with more mice demonstrated significantly lower immunogenicity in LPS pump-bearing mice immunized with the Spike encoding mRNA vaccine than in Saline pump-bearing mice (Supplementary Figure 4c–g). Overall, our findings indicate reduced immunogenicity in lymph nodes and splenocytes following mRNA vaccination in the mouse model of LPS-induced chronic inflammatory.

## Discussion

In developing mRNA vaccines against various viral infectious diseases, investigating the impact of these vaccines on various underlying diseases accompanied by low and sustained levels of inflammation, such as obesity, is crucial. Therefore, in this study, we established an animal model that induced chronic inflammation only, excluding other factors related to each disease, and investigated the effects of mRNA vaccines in this model. Based on this concept, we successfully established a chronic inflammation model that induced persistent mild inflammation in the heart via LPS pump implantation. mRNA vaccine administration in this chronic inflammatory context aggravated cardiac damage and myocarditis, indicating potential exacerbation of cardiac damage by mRNA vaccines in the presence of chronic inflammation. Furthermore, T-cell activation by mRNA vaccines could be downregulated in chronic inflammation.

Our results showed that mRNA vaccine immunization may induce mild inflammation and inflammatory cytokine production in the heart and muscles. Moreover, intramuscular mRNA vaccine administration during the chronic inflammation induced by LPS pump implantation dramatically increased inflammatory cell infiltration and myocarditis (Fig. 3). Previous reports have shown that elderly patients with chronic diseases, such as diabetes mellitus, hypertension, and hypercholesterolemia, have a higher risk of COVID-19 infection^26^. However, no clear evidence exists on the mRNA vaccine immunization-induced myocarditis in elderly patients with chronic diseases. However, myocarditis has been reported to be significantly more frequent after the second vaccine dose in males and people younger than 30 years of age^27,28^. The heightened risk of myocarditis associated with the two mRNA vaccines was evident only in individuals younger than forty years of age^5^. Moreover, previous studies have reported adverse effects of mRNA vaccines in the context of pre-existing inflammatory conditions, with growing concern regarding their safety profiles in individuals with underlying inflammatory disorders^14^. Consistently, in our chronic inflammatory mouse model, the enhanced cardiac damage and myocarditis following mRNA vaccine administration raises important questions regarding the potential risks of mRNA vaccines in individuals with pre-existing inflammatory conditions. Thus, the inflammatory milieu created by chronic inflammation may contribute to cardiac damage exacerbation following mRNA vaccine immunization.

Damaged skeletal muscles have an inherent capacity for self-regeneration and repair through myogenesis^29^. Notably, the regenerative potential of skeletal muscle tends to wane with increasing age and in chronic inflammation^30^. Localized inflammation at the injection site of COVID-19 mRNA vaccines has been documented in the muscles, with associated muscle damage^31,32^. Additionally, COVID-19 can cause muscle inflammation, and myositis has been noted alongside myopericarditis^33^. Here, we observed a significant increase in differentiation markers that characterize post-immunization muscle regeneration, suggesting the occurrence of muscle damage. Furthermore, alterations in the expression of factors related to mitochondrial fusion and fission were identified, indicating the potential involvement of mitochondria-derived damage-associated molecular patterns in affected muscles^34^. Damaged skeletal muscles trigger an immediate inflammatory response in the mitochondria, which is essential for myofibril regeneration. Notably, despite inducing an inflammatory response at the injection site, mRNA vaccines do not fully activate muscle repair signals. This observation was supported by the lack of significant changes in creatine kinase activity and a minimal increase in the number of regenerating muscle fibers and blood vessels. These alterations were less pronounced in the chronic inflammation group, indicating a diminished inflammatory response upon mRNA vaccination in chronic inflammation conditions. Whether the symptoms of myocarditis, despite a mild local reaction at the injection site, result from an innate immune response originating from immune cells within the muscle remains to be determined.

Additionally, we expanded our investigation beyond the cardiac and muscular effects of mRNA vaccines to explore the impact of chronic inflammation on the immunogenicity of these vaccines. We assessed both humoral and cellular immune responses to understand better the systemic immune responses in the presence of chronic inflammation. Humoral immune responses, which involve antibody production, are crucial for neutralizing pathogens and providing long-term protection against infections^35^. Notably, our analysis revealed that chronic inflammation induced by LPS did not significantly affect the humoral immune response to the mRNA vaccine. Preserving humoral immune responses in chronic inflammation suggests that antibody production and functionality remain intact following mRNA vaccine administration. Consistent with our results, previous studies have demonstrated that low-level inflammation induced by obesity does not affect the production of spike protein-specific IgG^6,36^.

In contrast to humoral immune responses, cell-mediated immune responses, specifically the number of splenocytes secreting IFN-γ in response to antigen stimulation, were lower in chronic inflammation. IFN-γ is a key cytokine produced by T cells and plays a crucial role in activating immune responses and promoting antiviral defense^37^. The reduction in IFN-γ-secreting splenocytes suggests a dampened T-cell immune response in chronic inflammation. The finding of diminished IFN-γ production aligns with previous research on the impact of chronic inflammation on T-cell function^38^. Chronic inflammation can cause T-cell exhaustion, characterized by the loss of effector functions and reduced cytokine production^39^. T-cell exhaustion is a mechanism that helps prevent excessive tissue damage during chronic inflammation but may compromise the immune response to pathogens or vaccines^38^.

Furthermore, the population of pro-inflammatory TNF-α and cytotoxic-granule granzyme B-positive CD8 T cells was lower in the chronic inflammation group. TNF-α is a pro-inflammatory cytokine that plays a central role in immune responses. Meanwhile, cytotoxic-granule granzyme B-positive CD8 T cells are effector T cells involved in eliminating infected cells^40,41^. Thus, the decreased pro-inflammatory and cytotoxic T-cell populations suggest T-cell exhaustion in chronic inflammation. These findings improve our understanding of the complex interplay between chronic inflammation and the immune response to mRNA vaccines. Chronic inflammation-induced T-cell exhaustion may impair the generation of robust and functional T-cell responses following mRNA vaccine administration. This compromised cell-mediated immune response may affect the effectiveness of mRNA vaccines in individuals with pre-existing inflammatory conditions.

In the context of the immunosuppressed patients, SARS-CoV-2 mRNA vaccines generated antibodies without significant side effects or disease flares, indicating they were efficient and safe in this population^42,43^. However, the delayed and reduced immune responses to SARS-CoV-2 in patients with immune-mediated inflammatory diseases (IMID) pose a challenge^14^. Insufficient data on how specific IMIDs and their treatments affect vaccine responses underscores the imperative for additional research in this domain. The compromised cell-mediated immune response observed in our study raises concerns about the potential impact on the efficacy of mRNA vaccines in individuals with pre-existing inflammatory conditions. Zirkenbach et al. demonstrated that mRNA vaccine administration increased myocarditis due to the activation of the immune response when immune checkpoint inhibitor (ICI) is administered as immunotherapy to cancer patients^44^. Consistently, our results indicated an increase in cardiac inflammation only in the environment of pre-conditioned chronic inflammation under mRNA vaccine administration. Meanwhile, another report indicated an increase in the free spike protein, unbound to antibodies, in vaccinated individuals who developed myocarditis^45^ suggesting potential evasion of antibody recognition. Individuals prone to myocarditis may have different innate immune responses, even if they receive the same vaccine. Additionally, T-cell involvement in postvaccine myocarditis cannot be conclusively ruled out because a slight increase in PD-1–expressing bulk CD4 + T cells suggests potential T-cell activation or exhaustion^45^. In contrast, our results showed no alterations in the expression of the spike protein and the production of antibodies against it in each organ, indicating that the innate immune response induced by the mRNA vaccine remained consistent. The question may arise about how mRNA vaccines trigger cardiac inflammation in these conditions. Intriguingly, our results showed that vaccination with mRNA vaccine in a chronic inflammatory environment resulted in T-cell exhaustion. It is possibly attributed to the mRNA vaccine intensifying the immune response in an environment characterized by heightened basal inflammation resulting from chronic inflammation, exceeding the immune capacity essential for maintaining immune system balance.

Our study offers insights into the impact of chronic inflammation on the toxicity and immunogenicity of mRNA vaccines. We found that chronic inflammation increases the risk of adverse events induced by mRNA vaccines, particularly in the heart, while the effects on skeletal muscles were less pronounced. Notably, the humoral immune response remained largely unaffected by chronic inflammation; however, the cell-mediated immune response was reduced. In conclusion, our findings suggest that cardiotoxicity in patients with chronic inflammation may occur during the immunogenic process of mRNA vaccines, which may be associated with reduced T-cell activation. Further research is thus warranted to elucidate the underlying mechanisms and assess the cardiac toxicity of mRNA vaccines in vulnerable populations with chronic inflammatory disorders.

## Methods

### Preparation of mRNA

The antigen was designed using the DNA sequence encoding the spike protein of the SARS-CoV-2 Omicron variant. The mRNA vaccine plasmid was produced by inserting antigen DNA into multiple cloning sites on the mRNA platform using restriction enzymes (Pac1 and Cla1), as previously described^46^. After the mRNA vaccine template was linearized using Not1, mRNA was produced using the EZ T7 High Yield In vitro Transcription Kit (Enzynomics, Daejeon, Korea) according to the manufacturer’s protocol. Capping was performed using SC101 (STPharm, Siheung, Korea), and UTP was replaced with N1-methyl-pseudouridine (Trilink, San Diego, CA, USA). Total mRNA was precipitated using lithium chloride and purified using cellulose, as described previously^47^.

### Lipid nanoparticle formulation for the mRNA vaccine

Lipid nanoparticles (LNPs) were prepared according to a reported protocol^48^. Briefly, all lipid components were dissolved in ethanol at a molar ratio of 50:10:38.5:1.5 (SM-102; distearoylphosphatidylcholine cholesterol, 1,2-dimyristoyl-rac-glycero-3-methoxypolyethylene glycol-2000), and mRNAs were dissolved in sodium citrate buffer (50 mM; pH 4) solution at a charge ratio of N/P = 6. LNPs were formulated using NanoAssemblr® IgniteTM (Precision Nanosystems, Vancouver, Canada) by mixing aqueous and organic solutions at a ratio of 3:1 and a total flow rate of 10 mL/min. The LNP solution was concentrated by ultrafiltration using an Amicon Ultracentrifugal filter (UFC9030, Merck Millipore, Billerica, MA, USA), following the manufacturer’s instructions.

### Physicochemical characterization of mRNA vaccine

The size and Zeta potential of the mRNA vaccine were measured using a zetasizer with diluted mRNA vaccine in water (Zetasizer Nano Zs, Malvern Panalytical, Malvern, Worcestershire, UK). The encapsulation efficiency of LNP is determined by Ribogreen assay. Both Triton X-100 treated or untreated mRNA vaccines were incubated with Ribogreen reagent of Quant-it™ RiboGreen RNA Assay Kit (Thermofisher, Waltham, MA, USA, Cat. R11490). A microplate reader analyzed the fluorescence for the total and free mRNA amount. The following formula was used to obtain the encapsulation efficiency of the mRNA vaccine:

$${\rm{Encapsulation\; efficiency}}\left( \% \right)=\left[\left({\rm{Total}}\,{\rm{mRNA}}-{\rm{free}}\,{\rm{mRNA}}\right)/{\rm{total}}\,{\rm{mRNA}}\right]\times 100 \%$$

The endotoxin level is determined to be lower than 0.1 EU/ml using the LAL assay kit (Cat. L00350, Genscript, Piscataway, NJ, USA).

### Cryo-TEM sample preparation and data collection

Three microliter of mRNA vaccine solution was loaded onto a holey carbon grid (Quantifoil R1.2/1.3, 200 mesh Cu, Structure Probe Inc., USA) treated with a glow discharger at 15 mA for 60 s to increase the loading efficiency. The grid was blotted for 4 s at 15 °C with 100% humidity using a Vitrobot Mark IV (Thermofisher, USA, SNU CMCI) and immediately plunge-frozen in liquid nitrogen-cooled liquid ethane. The grids were imaged with TEM (JEM-2100F, JEOL, Japan) while maintaining the temperature at ~−180 °C at an acceleration voltage of 200 keV. Images were recorded using an ultrascan 1000 electron detector.

### Establishment of the chronic inflammatory mouse model by implanting a pump containing LPS

Female Balb/c and C57BL6 mice at the age of 6–8 weeks were obtained from Dae-Han Biolink Co. (Eumseong, Korea) and housed in a controlled environment (inverted 12-h daylight cycle) with free access to food and water. All procedures were complied with the ARRIVE guidelines and approved by the Institutional Animal Care and Use Committee of the Samsung Biomedical Research Institute (#2022032201). The Samsung Biomedical Research Institute is accredited by the Association for Assessment and Accreditation of Laboratory Animal Care International and abides by the Institute of Laboratory Animal Resources guidelines. Mice were fed a normal-fat diet (containing 5% fat) and simultaneously treated with LPS from *Escherichia coli* (Sigma, St Louis, MO, USA). The surgical procedure for inserting the osmotic pump followed the manufacturer’s instructions. Briefly, after the hair was shaved, the back of the mouse was incised, and a space was made in the subcutaneous tissue. The device was inserted into the space, and the incision was closed using wound clips (7 mm). The mice were implanted with an osmotic pump (Alzet model 1004; DURECT Corp., Cupertino, CA, USA) filled with either Tween-saline (0.9% NaCl and 0.1% Tween 80 in distilled water, Sigma), normal saline control, or LPS diluted in Tween-saline infused at 300 µg/kg/day for 4 weeks (LPS group)^49^. For blood collection, mice were anesthetized with isoflurane (Isotroy 100, Troikaa, Gujarat, India), and blood was collected from the facial vein. At the defined endpoint (2 days after the boost), mice were euthanized by CO_2_ inhalation, and their spleens, lungs, blood, hearts, and muscles were harvested.

### Immunization

The pump-implanted animals were randomly assigned to four groups for administering the intramuscular SARS-CoV-2 mRNA vaccine or normal saline control (groups: saline pump, saline pump + mRNA vaccine, LPS pump, and LPS pump + mRNA vaccine). The mice were immunized intramuscularly with 10 µg of the Omicron S mRNA vaccine with the sequence encoding the spike protein from the SARS-CoV-2 Omicron variant. The mRNA expression platform has been previously described^22^. The immunization schedule comprised two injections: an initial prime injection followed by a booster injection, with a 2-week interval between injections. Once the immunization protocol was completed, the mice were euthanized 2 days after immunization, and whole blood samples and tissues were collected.

### Flow cytometric analysis

Splenocytes (1 × 10^6^) isolated from immunized mice were cultured in 96-well plates. Subsequently, the samples were treated with Brefeldin A (Golgi plug, BD Biosciences, Franklin Lakes, NY, USA) and stimulated with antigen peptides of the spike protein from the SARS-CoV-2 Omicron variant (5 μg/mL) in RPMI medium for 12 h at 37 °C. Then, the splenocytes were treated with anti-mouse CD16/32 (Invitrogen, Waltham, Massachusetts, USA) for 20 min at 4 °C. To stain surface proteins such as CD8, the splenocytes were stained with anti-mouse CD8 fluorescent antibody for 30 min at 4 °C in the dark. Then, the samples were fixed and permeabilized using BD Cytofix/Cytoperm™ (BD Biosciences), stained with anti-mouse tumor necrosis factor alpha (TNF-α) and granzyme B fluorescent antibody for 30 min at 4 °C in the dark. For detecting the levels of M1 and M2 macrophages in the lungs using flow cytometry, lung tissues excised from immunized mice were digested with Hanks’ Balanced Salt Solution containing 1.5 mg/ml collagenase A, 0.1 mg/ml Dnase1 (Sigma Aldrich, Burlington, Massachusetts, United States) for 1 h at 37 °C and stained using anti-mouse CD11b, CD11c, and F4/80 with the surface protein staining method used for splenocytes. The samples were then fixed and permeabilized using a Foxp3 Fixation Kit (Invitrogen) according to the manufacturer’s instructions. The cells were stained with anti-mouse CD206 CD8 fluorescent antibody in the dark for 30 min at 4 °C. The samples were analyzed using a CytoFlex flow cytometer (Beckman Coulter, Brea, CA, USA), and data were analyzed using CytExpert (Beckman Coulter, Brea, CA, USA).

### Enzyme-linked immunosorbent assay (ELISA)

Enzyme-linked immunosorbent assays (ELISAs) were performed to assess antigen-specific total IgG levels in mouse serum. Briefly, a 96-well plate was coated with S protein from the SARS-CoV-2 Omicron variant at a concentration of 100 ng/well and incubated overnight at 4 °C. The wells were then blocked with 100 µl blocking buffer (1% BSA in PBS) for 1 h at room temperature. The serum samples diluted with blocking buffer (at 1:20) were added to the wells and incubated for 2 h at room temperature (20–22 °C). After the incubation period, the wells were washed thrice with 200 µl PBS-T (PBS containing Tween 20). Horseradish peroxidase-conjugated anti-mouse IgG antibodies (from Bethyl Laboratories, Montgomery, TX, USA) were added to the wells and then the plates were incubated at room temperature for 1 h. The antibodies were appropriately diluted in blocking buffer (at 1:5000). Following three washes with PBS-T, tetramethylbenzidine substrate was added to the wells, and the plates were incubated for 15 min. The reaction was stopped by adding 2 N H_2_SO_4_. Finally, the optical density was measured at 450 nm using a microplate reader (GloMax Explorer, Promega, Seoul, Republic of Korea).

### Enzyme-linked immunospot (ELISpot) assay

Splenocytes (5 × 10^5^) isolated from immunized mice were cultured in 96-well MultiScreen-IP Filter Plates (Millipore, Burlington, MA, USA) and stimulated with antigen peptides of spike protein from the Wuhan SARS-CoV-2 strain (5 μg/ml) in RPMI medium for 24 h at 37 °C. The ELISpot assay for detecting IFN-γ secreted from splenocytes was performed as per the manufacturer’s instructions (Mab-tech, Stockholm, Sweden).

### Histological analysis

The epididymal fat, liver, lung, muscle, and pancreatic tissues of osmotic pump-implanted mice were fixed in 10% neutral formalin. After fixation, the samples were embedded in paraffin and stained with hematoxylin and eosin (H&E). The percentage area of myocardial inflammation was determined using computer-assisted analysis. Two different areas of each heart were quantified using ImageJ software1.45 s (NIH, MA, USA), as described previously^50^. To analyze the damaged muscle area, a Motic EasyScan Digital Slide Scanner (Motic Hong Kong Ltd., Hong Kong, China) was used to randomly capture 5–10 images of the affected regions. These images were then analyzed for the cross-sectional area (CSA) of the centrally nucleated regenerating myofibers using ImageJ software^51^. The procedures were performed by investigators who were blinded to the identities of the samples. In the SARS-CoV-2 spike immunohistochemistry procedure, Dako Retrieval Solution (pH 6.0, S2369) was utilized for antigen retrieval through incubation of sections. To mitigate nonspecific binding, a blocking step of 1-hour duration was carried out using Dako Protein Block Serum-Free (X0909). Subsequently, the sections were exposed to anti-SARS-CoV/SARS-CoV-2 (COVID-19) spike antibody (1:200; GTX632604, GeneTex) for 24 h at 4 °C. The visualization of antigens was enabled using the Dako Envision Detection system Peroxidase/DAB+ (K5007). Following this, the slides were dehydrated and mounted after counterstaining with hematoxylin. Images of the SARS-CoV-2 spike protein were captured randomly using a Motic Easyscan Digital Slide Scanner (Motic Hong Kong Limited).

### Quantitative reverse transcription-PCR

Total RNA was extracted from lung tissue using the TRIzol reagent (Invitrogen, Grand Island, NY, USA), and cDNA was synthesized using a cDNA synthesis kit (Applied Biosystems, Foster City, CA, USA) according to the manufacturer’s instructions. Real-time PCR was performed using the SYBR Green Master Mix (Applied Biosystems, Foster City, CA, USA) to compare the mRNA levels of genes associated with inflammation, myogenesis, muscle types, and mitochondria. The primer sets were synthesized by SFC-probe (Cheongju, Korea) for inflammation-related genes and Bioneer (Daejeon, Korea) for the remaining genes. The primer sequences are listed in Supplementary Table 1.

### Creatine kinase assay

Plasma was isolated from mouse blood and processed according to the manufacturer’s protocol for the Creatine Kinase Activity Assay Kit (3050 Spruce Street, St. Louis, MO, USA). The creatine kinase activity was determined by measuring absorbance (340 nm) using a VICTOR Nivo ^TM^ multimode Microplate Reader (PerkinElmer, Waltham, MA, USA) following the standard procedure for assessment.

### Statistical analysis

Statistical analyses were performed using Prism 8 software (GraphPad, San Diego, CA, USA). Data were presented as mean ± standard deviation. Significant differences between means were determined using Student’s *t*-test, and *P* values less than 0.05 were considered statistically significant.

### Reporting summary

Further information on research design is available in the Nature Research Reporting Summary linked to this article.

### Supplementary information

Supplementary information

REPORTING SUMMARY

## Data Availability

All data upon which conclusions are drawn are included in the manuscript or in the supplemental information file provided.
