# Supplementary material for: Assessing the impact of mRNA vaccination in chronic inflammatory murine model
Source: NPJ Vaccines. 2024 Feb 15;9:34. doi: 10.1038/s41541-024-00825-z (PMC10869740; doi:10.1038/s41541-024-00825-z)
Supplement: Supplementary file 1 — Supplementary information [file 41541_2024_825_MOESM1_ESM.pdf]

SUPPLEMENTARY TABLE, FIGURES

Supplementary Table 1. Sequences of the primers used in the study

Tissue	Gene	Forward (5′-3′)	Reverse (5′-3′)
Heart	IL-1β	TTGACGGACCCCAAAGAGTG	ACTCCTGTACTCGTGGAAGA
	IL-6	GTACTCCAGAAGACCAGAGG	TGCTGGTGACAACCACGGCC
	TNF-α	TTGACCTCAGCGCTGAGTTG	CCTGTAGCCCACGTCGTAGC
	CD4	GTTCAGGACAGCGACTTCTGGA	GAAGGAGAACTCCGCTGACTCT
	CD8	ACTACCAAGCCAGTGCTGCGAA	ATCACAGGCGAAGTCCAATCCG
	MCP-1	ACCTGGATCGGAACCAAATG	CCTTAGGGCAGATGCAGTTTTAA
	NPPA	TACAGTGCGGTGTCCAACACAG	TGCTTCCTCAGTCTGCTCACTC
	Myh7	GCTGAAAGCAGAAAGAGATTATC	TGGAGTTCTTCTCTTCTGGAG
	ANP	TCGTCTTGGCCTTTTGGCT	TCCAGGTGGTCTAGCAGGTTCT
	GAPDH	ATCAACGACCCCTTCATTGACC	CCAGTAGACTCCACGACATACTCAGC
Muscle	Adgre1	GCTGCACCTCTGTGCCTTT	CAGGTATGCCATGATGCTTG
	CD80	CGCAACCACACCATTAAG	GATGACGACGACTGTTATTAC
	IL-1β	GCAACTGTTCCTGAACTCAACT	ATCTTTTGGGGTCCGTCAACT
	CD206	GAGTGGCAGGTGGCTTATG	ATTTGGGTTCAGGAGTTGTTGT
	Myh7	ACCAGGCCCTTTGACCTCAAGAAA	TCTTGTCGAACTTGGGTGGGTTCT
	MyoD	AGACTTCTATGATGACCCGTGTT	TCAGCGTTGGTGGTCTTGC
	IGF- II	CGCTTCAGTTTGTCTGTTCG	AGGTAGACACGTCCCTCTCG
	GAPDH	TCCCACTCTTCCACCTTCGA	CAGGAAATGAGCTTGACAAAGTTG
	ND1	CTAGCAGAAACAAACCGGGC	CCGGCTGCGTATTCTACGTT
	HK2	GCCAGCCTCTCCTGATTTTAGTGT	GGGAACACAAAAGACCTCTTCTGG
	PGC-1α	CGGAAATCATATCCAACCAG	TGAGGACCGCTAGCAAGTTTG
	Opa1	CGACTTTGCCGAGGATAGCTT	CGTTGTGAACACACTGCTCTTG
	Drp1	CCTCAGATCGTCGTAGTGGGA	GTTCCTCTGGGAAGAAGGTCC
	Mfn1	AACTTGATCGAATAGCATCCGAG	GCATTGCATTGATGACAGAGC
	Mfn2	CTGGGGACCGGATCTTCTTC	CTGCCTCTCGAAATTCTGAAACT
	TNNC1	GCGGTAGAACAGTTGACAGAG	CCAGCTCCTTGGTGCTGAT
	TNNC2	GAGGCCAGGTCCTACCTCAG	GGTGCCCAACTCTTTAACGCT
	TNNI1	ATGCCGGAAGTTGAGAGGAAA	TCCGAGAGGTAACGCACCTT
	TNNI2	CGGAGGGTGCGTATGTCTG	CAGGTCCCGTTCCTTCTCA

Abbreviations: IL-1β, Interleukin 1 beta; IL-6, Interleukin 6; TNF-a, tumor necrosis factor-alpha; MCP-1, monocyte chemoattractant protein-1; monocyte chemoattractant protein-1; NPPA, natriuretic peptide A; Myh7, myosin heavy chain 7; ANP, atrial natriuretic peptide; GAPDH, glyceraldehyde-3-phosphoate dehydrogenase; MyoD, Myogenic Differentiation 1; IGF- II, Insulin Like Growth Factor 2; ND1, NADH dehydrogenase subunit 1; HK2, hexokinase 2; PGC-1α, Peroxisome Proliferator-Activated Receptor Gamma Coactivator 1-Alpha; Opa1, Optic atrophy protein 1; Drp1, Dynamin-Related Protein 1; Mfn1, Mitofusin 1; Mfn2, Mitofusin 2; TNNC1, Troponin C Type 1; TNNC2, Troponin C Type 2; TNNI1, Troponin I1; TNNI2, Troponin I2

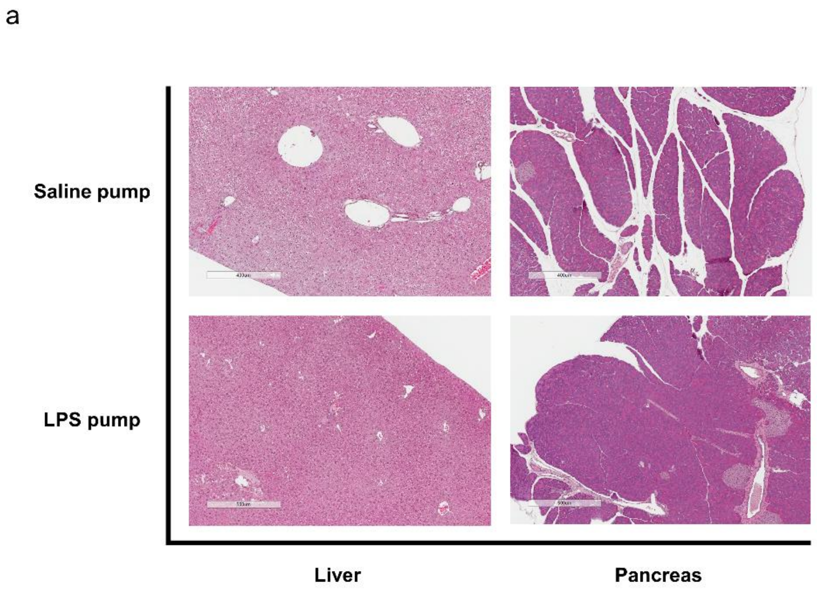

Supplementary Figure 1. Histological findings of the liver and pancreas. No difference was found in tissue inflammation and damage upon lipopolysaccharide pump implantation. LPS, lipopolysaccharide. Each scale bars indicate 400 μm, 500 μm (Saline pump, LPS pump).

a

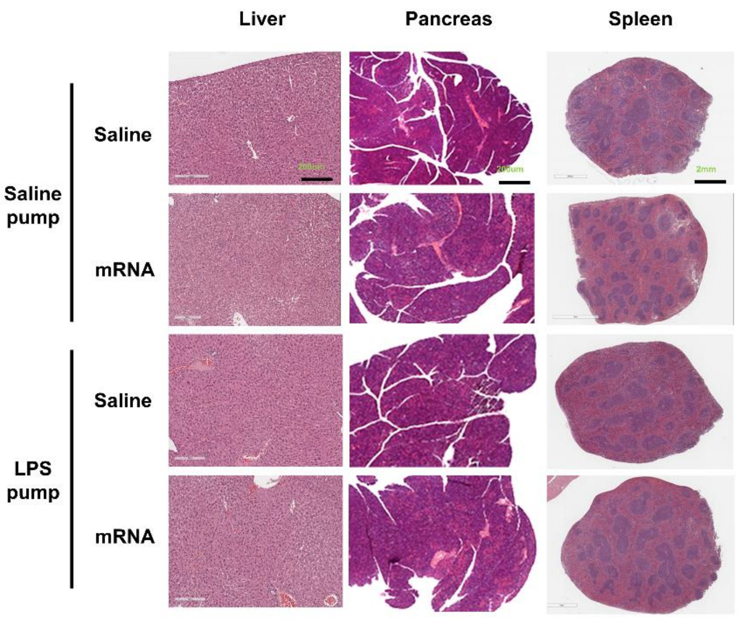

b

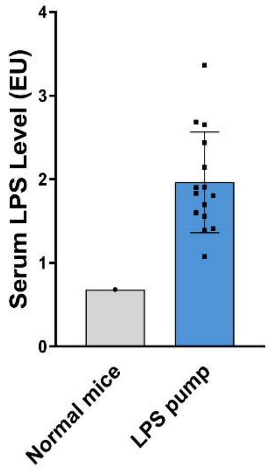

Supplementary Figure 2. (a) Histological findings of the liver, pancreas, and spleen. Each scale bars indicate 200 μm, 200 μm, 2 mm (Liver, pancreas, spleen). No difference was found in tissue inflammation and damage upon lipopolysaccharide (LPS) pump implantation with or

without mRNA vaccine administration. (b) Serum LPS levels were measured using enzyme-linked immunosorbent assay.

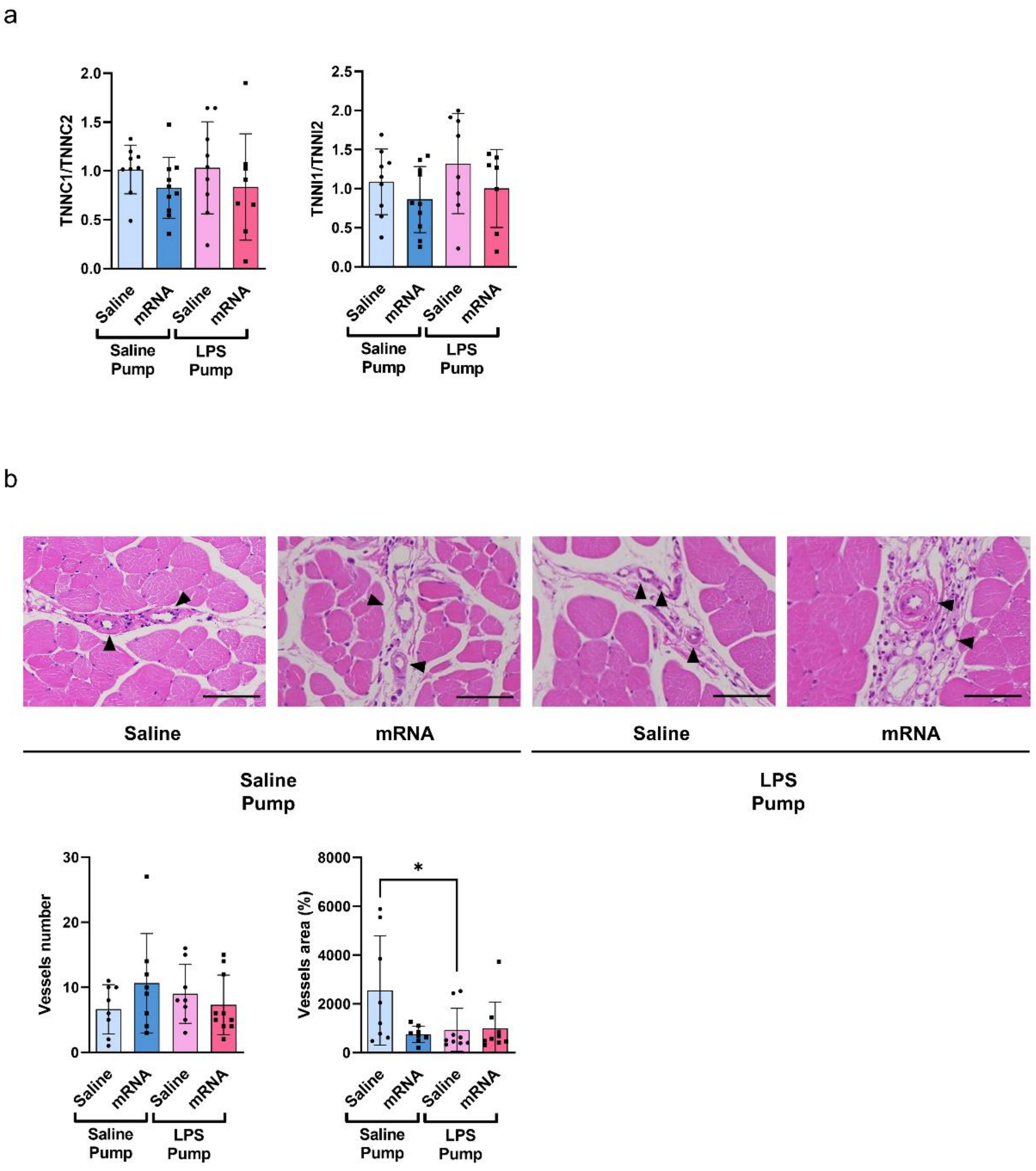

Supplementary Figure 3. mRNA vaccines do not induce significant adverse effects in the muscle. (a) The quadriceps were obtained two days after administering an immunization booster and were analyzed using quantitative reverse transcription PCR (n = 7–10). The reference gene, mouse GAPDH, was used to normalize the target gene expression levels. (b) Representative hematoxylin and eosin images of muscles from each group are shown (scale

bar = 60 µm). The vessels in the quadricep muscles were counted, and their respective areas were measured. *$P < 0.05$ by a two-tailed Student's t-test. All data are presented as the mean ± standard deviation. LPS, lipopolysaccharide.

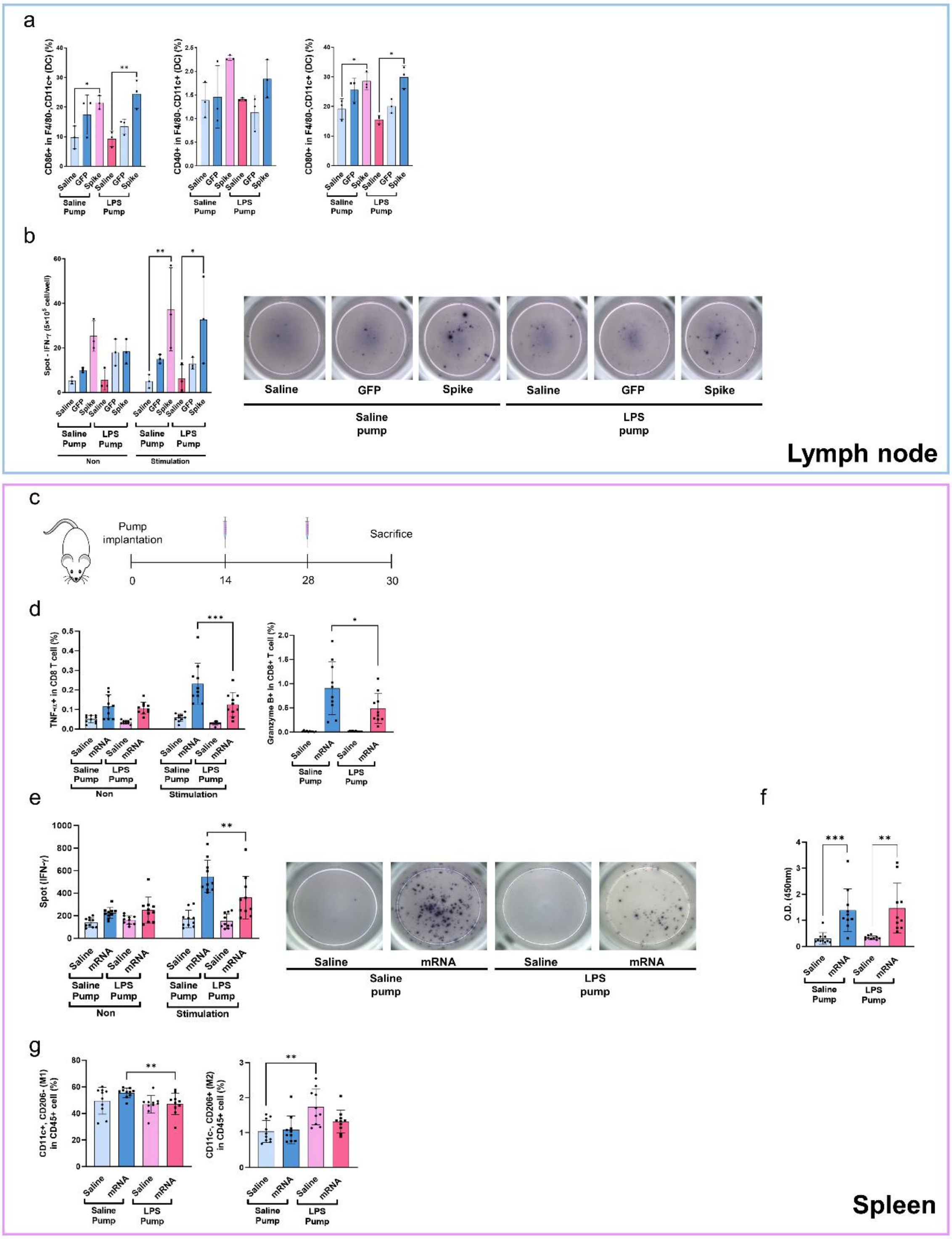

Supplementary Figure 4. Effects of chronic inflammation on the immunogenicity of mRNA vaccines. (a) The activation levels in dendritic cells in lymph nodes were analyzed with flow cytometry. (b) The IFN-γ secreting cell numbers were determined using ELISpot in the

lymph node. (c) C57BL/6 mice were intramuscularly primed and boosted with lipid nanoparticle-formulated mRNA-Omicron (10 μg) at 2-week intervals and were then sacrificed 2 days after boosting. (d) Percentages of tumor necrosis factor-alpha- and granzyme B-producing $CD4^+$ and $CD8^+$ T cells in the spleen were assessed using flow cytometry. (e) Enzyme-linked immunospot (ELISpot) assay for interferon-gamma (IFN-γ) produces splenocyte activity. Splenocytes were stimulated for 2 days with/without Omicron-specific T-cell peptide. The IFN-γ secreting cell numbers were determined using ELISpot. (f) Omicron-specific IgG levels were measured using enzyme-linked immunosorbent assay. Data are presented as mean ± standard deviation. Statistical significance was analyzed using a two-tailed Student's *t*-test. The significance of differences between the groups is indicated on the bars: *$P < 0.05$, **$P < 0.01$, ***$P < 0.005$ *vs.* the saline pump or mRNA vaccination group. IFN- γ, interferon-gamma; LPS, lipopolysaccharide; TNF-α, tumor necrosis factor alpha. (g) The mRNA vaccine slightly induced immunosuppressive responses in the lungs of the chronic inflammation-induced mice. Data are presented as the mean ± standard dev iation. Statistical significance was analyzed using a two-tailed Student's *t*-test. The significance of differences between groups is indicated on the bars: *$P < 0.05$, ** $P < 0.01$, ***$P < 0.005$ by a two-tailed Student's t-test (a, d-right, e) or two-way ANOVA (b, d-left, f, g). *vs*. the saline-pump or mRNA vaccination group. LPS, lipopolysaccharide.

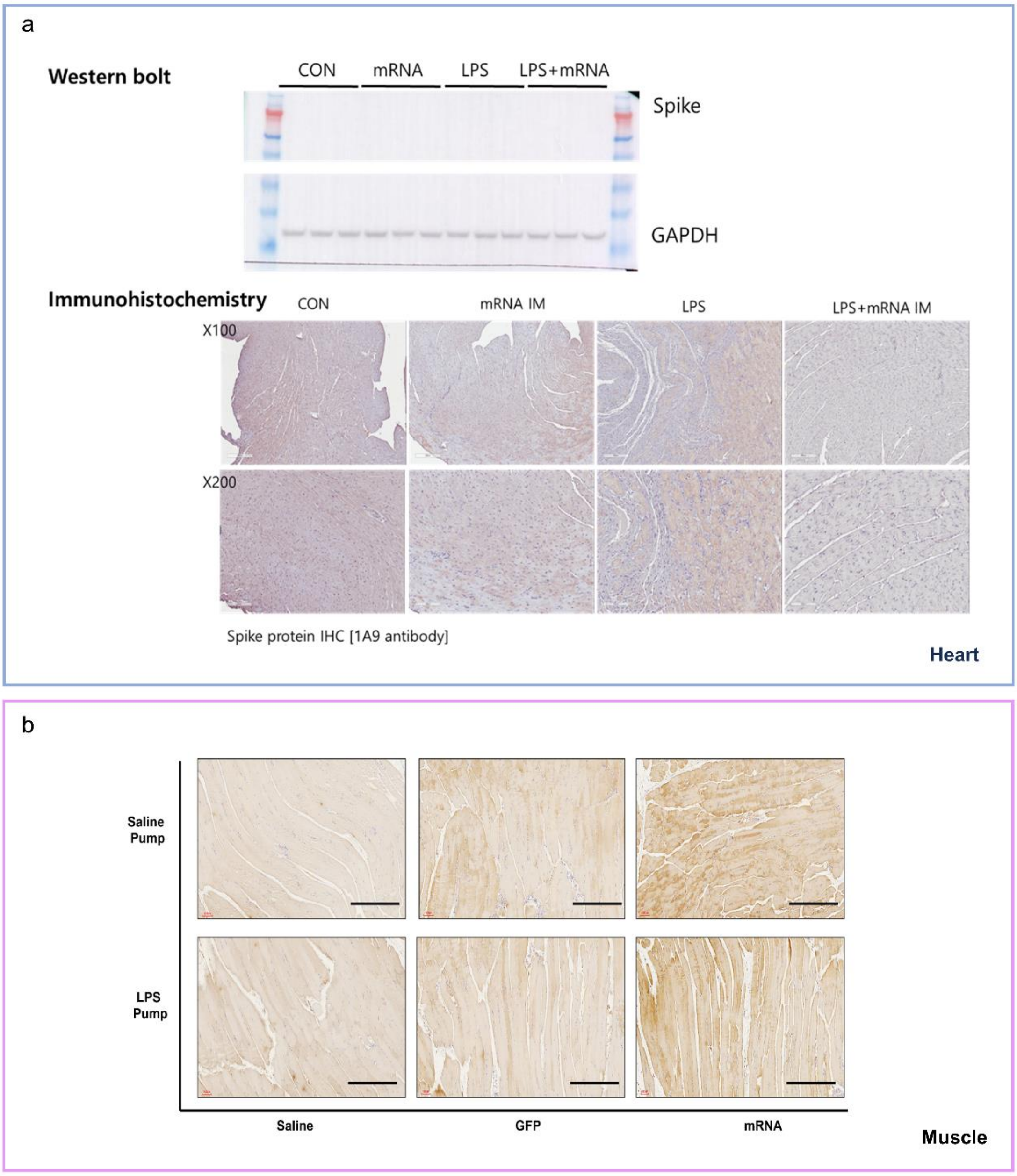

Supplementary Figure 5. Effects of chronic inflammation on spike protein expression by the mRNA vaccines. (a) The protein was extracted from the heart tissue of mice subjected to mRNA vaccine administration. Protein and heart sections were subjected to western blot analysis and immunohistochemistry using the SARS-COV2 spike antibody [1A9]. (Scale bar = 200 μm). (b) Muscle tissue sections were subjected to immunohistochemistry.

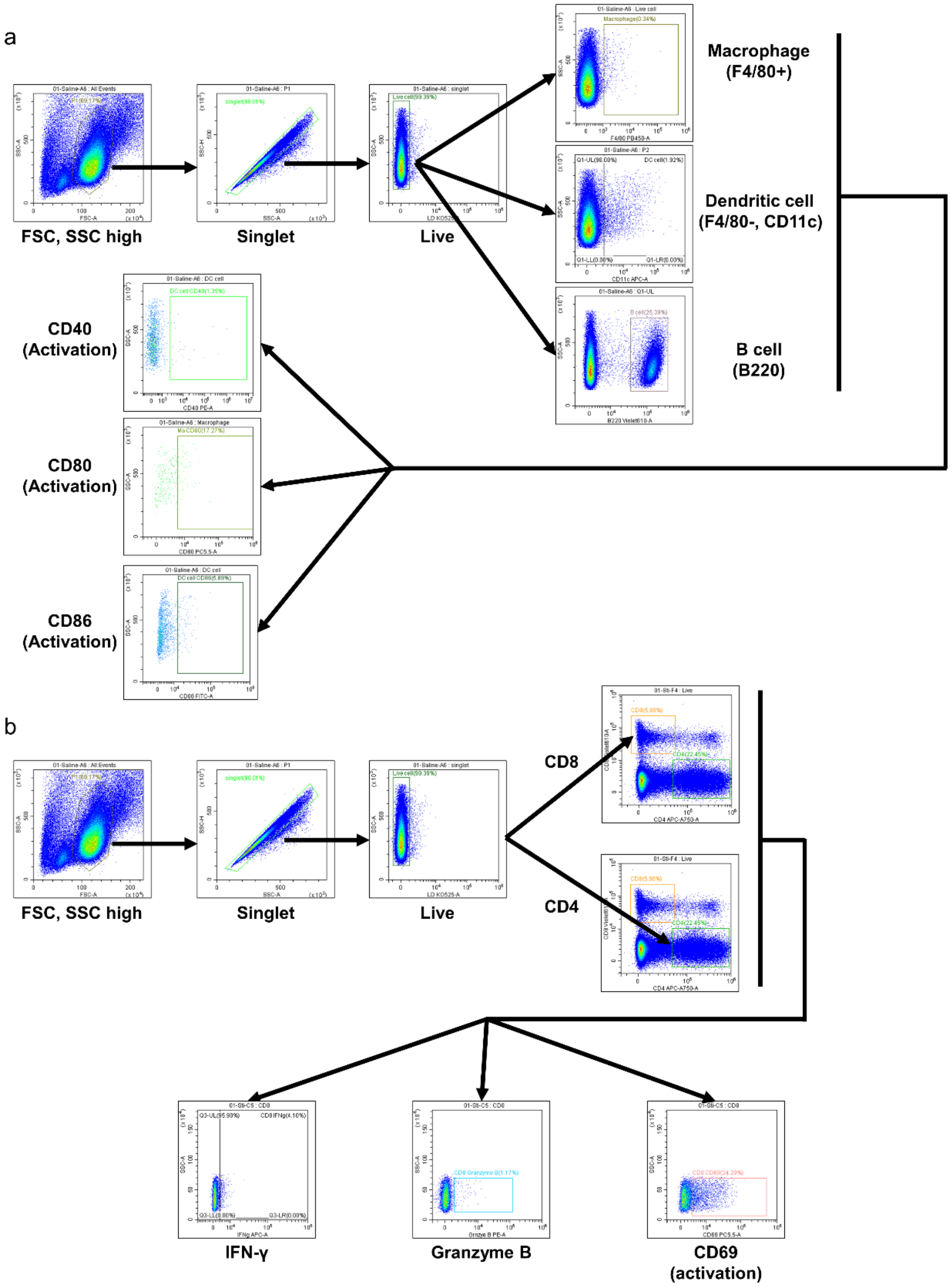

Supplementary Figure 6. (a) Flow cytometry plot of CD40, CD80, and CD86 (Antigen presenting cell activation marker) positive macrophages, dendritic cells, and B cells. (b) Flow cytometry plot of IFN-γ, Granzyme B, and CD69 (T cell activation marker) positive CD4 and CD8 T cells.
